# Systemic immune-inflammatory markers and long-term prognosis after revascularization in Moyamoya disease: a retrospective study

**DOI:** 10.3389/fneur.2024.1418729

**Published:** 2024-09-02

**Authors:** Shuang Wang, Wei Liu, Yuanren Zhai, Chenglong Liu, Peicong Ge, Dong Zhang

**Affiliations:** ^1^Department of Neurosurgery, Beijing Tiantan Hospital, Capital Medical University, Beijing, China; ^2^China National Clinical Research Center for Neurological Diseases, Beijing, China; ^3^Department of Neurosurgery, Beijing Hospital, National Center of Gerontology, Beijing, China; ^4^Institute of Geriatric Medicine, Chinese Academy of Medical Sciences, Beijing, China

**Keywords:** systemic immune-inflammatory markers, Moyamoya disease, long-term prognosis, lymphocyte-to-monocyte ratio (LMR), stroke

## Abstract

**Background:**

Systemic immune-inflammatory markers combine various individual inflammatory cell parameters to comprehensively explore their relationship with the development and long-term outcomes of cardiovascular, cerebrovascular, and oncological disorders. The systemic immune-inflammatory marker index has not been extensively studied in terms of its impact on the long-term prognosis following cerebral revascularization in MMD patients. Our research aims to address this gap and improve the prediction of long-term outcomes for these patients.

**Methods:**

We included 851 patients with Moyamoya disease who underwent cerebral revascularization at our medical center from 2009 to 2021. Systemic immune-inflammatory markers were calculated based on routine blood test results at admission, and follow-up was conducted for over 6 months after surgery. During monitoring and upon release, we evaluated patient neurological condition by utilizing the modified Rankin Scale (mRS). We examined the correlation between alterations in mRS ratings and systemic immune-inflammatory markers.

**Results:**

Comparing the unfavorable long-term prognosis group to the favorable long-term prognosis group, it was found that the NLR level was markedly higher (*p* = 0.037), while the LMR was lower in the unfavorable long-term prognosis group (*p* = 0.004). Results from logistic regression analysis revealed that the high-level LMR group had a lower risk of unfavorable long-term prognosis compared to the low-level group (T3: OR = 0.433, 95% CI [0.204–0.859], *p* = 0.026). The AUC of the model was 0.750 (95% CI [0.693–0.806]).

**Conclusion:**

Lymphocyte-to-monocyte ratio levels are independently linked to an increased risk of unfavorable long-term prognosis, highlighting LMR as a new and effective predictor for postoperative Moyamoya patients.

## Introduction

Moyamoya disease is a rare cerebrovascular condition characterized by progressive narrowing of the terminal segments of both internal carotid arteries. This leads to the formation of an abnormal vascular network with collateral circulation at the base of the brain, elevating the risk of ischemic or hemorrhagic stroke and potentially resulting in high morbidity and mortality rates (1). Moyamoya disease arises from a complex interaction of genetic, immune, inflammatory, and other factors. Several researches have emphasized the significant impact of persistent systemic inflammation on the development of Moyamoya disease (2). Regarding treatment, cerebral revascularization surgery is widely regarded as the most effective approach, which encompasses direct, indirect, and combined bypass techniques (3–5). Previous studies have indicated that advanced Suzuki stage, RNF213 p.R4810K variant, diabetes, and the nutritional index (PNI) are risk factors associated with long-term unfavorable long-term prognosis following cerebral revascularization (6–9). However, the factors influencing the long-term prognosis of patients with Moyamoya disease (MMD) after cerebral revascularization are still not fully elucidated.

Neutrophil-to-lymphocyte ratio (NLR), platelet-to-lymphocyte ratio (PLR), systemic immune-inflammatory index (SII), and lymphocyte-to-monocyte ratio (LMR) are some readily accessible inflammatory markers in clinical practice. They are more indicative of systemic inflammation levels compared to standard neutrophil (Neut) or lymphocyte (Ly) counts alone. The NLR, PLR, LMR, and SII have been extensively employed in forecasting the long-term outcomes in individuals with different types of cancer such as breast cancer, hepatocellular carcinoma, stomach cancer, and tongue cancer (10–12). Recent research has shown that the neutrophil-to-lymphocyte ratio (NLR) can be a reliable indicator of poor long-term outcomes for individuals with cardiovascular conditions (13). Additionally, there has been a growing body of evidence suggesting a connection between markers of systemic immune inflammation and the likelihood of developing Moyamoya disease (MMD). These findings have significant implications for the management and treatment of patients with these health concerns (14). This study aims to explore the potential relationship between systemic immune-inflammatory marker levels upon admission and the long-term prognosis after cerebral revascularization surgery in patients with Moyamoya disease (MMD), and to establish an appropriate predictive model.

## Methods

### Study design and participants

**Figure 1:**
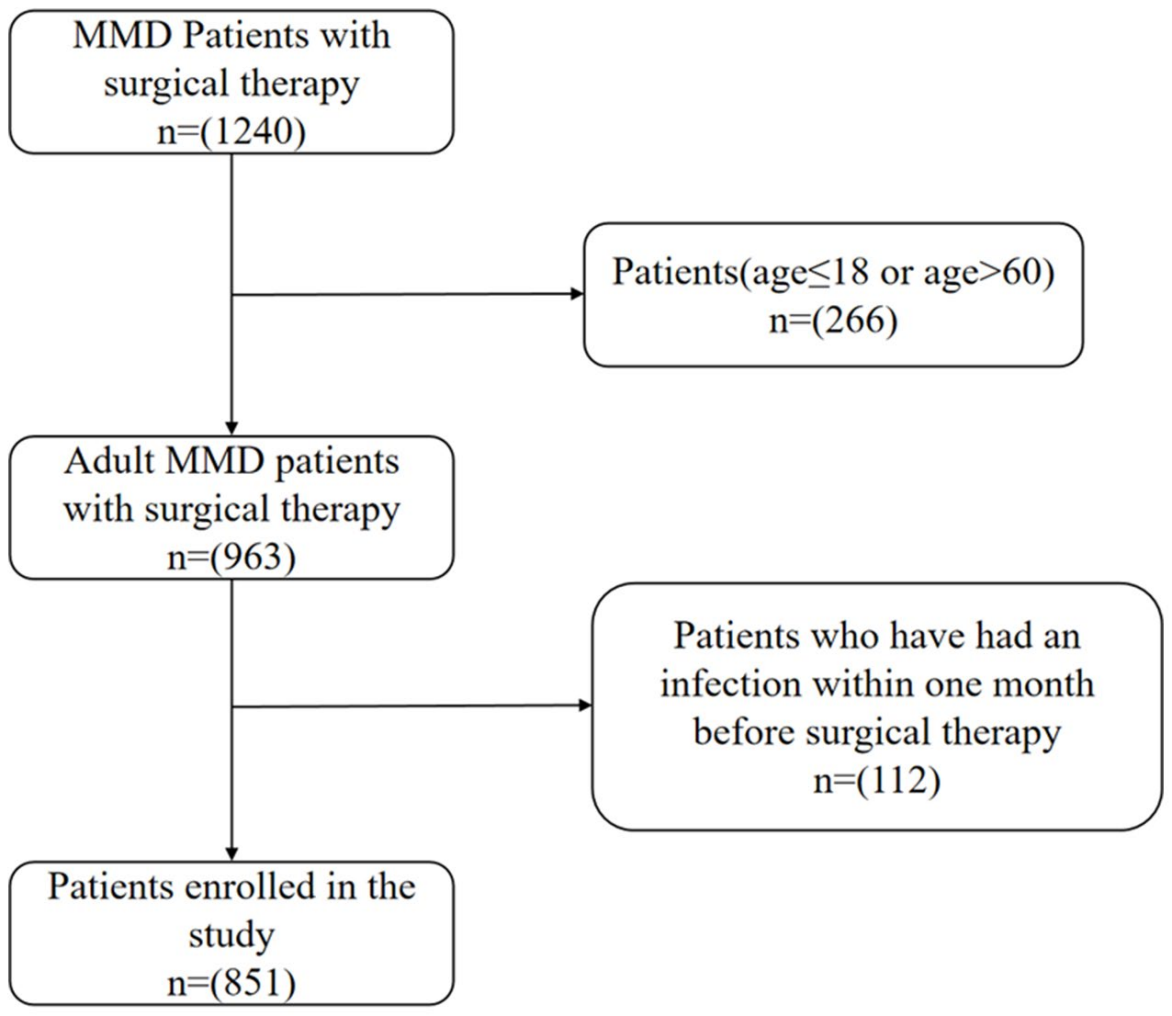
Flow diagram of the study participants. MMD indicates moyamoya disease.Infection is defined as a white blood cell (WBC) count greater than 10.0 ×10^9^/L.

We conducted a retrospective analysis of patients with Moyamoya disease (MMD) who visited Beijing Tiantan Hospital from 2009 to 2021 and underwent cerebral revascularization surgery. Confirmation of Moyamoya disease (MMD) was established through the utilization of digital subtraction angiography (DSA) as outlined in the 2012 Japanese guidelines. The criteria for exclusion were as follows: (1) individuals below the age of 18 or over the age of 60; (2) patients who have had a clearly identified source of infection within 1 month before admission, or cases where the source of infection is unclear but the white blood cell count is greater than or equal to 10 × 10^9^/L; (3) patients with secondary moyamoya syndrome, including those with concurrent conditions such as atherosclerosis, vasculitis, neurofibromatosis, autoimmune diseases, and other metabolic diseases; (4) 28 patients lost to follow-up were excluded. Finally, 851 patients with MMD were included in our study, including 765 in the favorable long-term prognosis group and 86 in the unfavorable long-term prognosis group. The Ethics Committee at Beijing Tiantan Hospital approved all study protocols (Approval Number: KY2016-048-01) in accordance with the Helsinki Declaration principles. This research is recorded in the Chinese Clinical Trial Registry (ChiCTR2000031412). Informed consent was obtained from all participants (Figure 1).

#### Baseline data collection

We collected baseline data from all participants, including age, gender, RNF213 p.R4810K variant, body mass index (BMI), Suzuki stage, surgical modality, and medical history, such as diabetes, hypertension, dyslipidemia, smoking, and alcohol consumption. We define Suzuki stages 1–3 as early stage and stages 4–6 as advanced stage. Based on the clinical phenotypes, patients were categorized into groups of transient ischemic attack (TIA), infarction, or hemorrhage. On the morning of the operation, peripheral blood samples were collected from all MMD patients after fasting for more than 12 h and analyzed using an automated hematology analyzer to measure white blood cells (WBC), lymphocytes (LY), neutrophils (NEUT), monocytes (MONO), platelets (PLT), alanine aminotransferase (ALT), aspartate aminotransferase (AST), Calcium (Ca), Phosphorus(P), triglycerides (TG), total cholesterol (CHO), high-density lipoprotein cholesterol (HDL-C), low-density lipoprotein cholesterol (LDL-C), apolipoprotein A1 (ApoA1), apolipoprotein B (ApoB), and homocysteine (Hcy). Systemic immune-inflammatory markers, including neutrophil-to-lymphocyte ratio (NLR), platelet-to-lymphocyte ratio (PLR), lymphocyte-to-monocyte ratio (LMR), and systemic immune-inflammatory index (SII), were calculated as described previously.

### Treatment and changes in long-term prognosis

Our neurosurgical team performed three types of cerebral revascularization surgeries on patients: direct, indirect, and combined bypass. At our medical center, direct and combined bypass techniques were the preferred methods, with indirect bypass being selected in instances where the superficial temporal artery or middle cerebral artery was deemed too delicate for bypass surgery. Clinical presentation of patients was the main consideration for cerebral revascularization surgery, focusing on symptomatic hemispheres of the brain. Long-term prognosis were evaluated through face-to-face or telephone interviews conducted after discharge, with a median follow-up time of 29.27 months. Follow-up events included transient ischemic attack (TIA), ischemic stroke, and hemorrhagic stroke. We assessed patients’ neurological status using the modified Rankin Scale (mRS), where scores of 0–2 indicate favorable neurological status and 3–5 indicate unfavorable status. Two neurosurgery residents evaluated mRS scores of patients without knowledge of the surgical side or systemic immune-inflammatory markers and compared them with the mRS scores upon discharge. A stable or decreased mRS score compared to discharge was considered a favorable long-term prognosis, while an increased score was considered a unfavorable long-term prognosis.

### Statistical analysis

We performed statistical analysis and graphical display using SPSS version 25.0 (IBM Corporation, Armonk, NY, United States), Empower(R) (www.empowerstats.com, X&Y Solutions, Inc., Boston, MA, United States), and Prism (GraphPad Prism Version 10.0 for Windows, GraphPad Software, Boston, MA, United States, www.graphpad). A two-tailed test with *p* < 0.05 was considered statistically significant. Baseline differences between the favorable and unfavorable long-term prognosis groups of patients with Moyamoya disease (MMD) were compared. Continuous variables were expressed as mean ± standard deviation (SD) or median and interquartile range (IQR), while categorical variables were expressed as frequencies. All participants were divided into three groups according to tertiles of systemic immune inflammatory markers: NLR (T1 ≤ 1.718, T2:1.718 to < 2.312, T3 ≥ 2.312); PLR (T1 ≤ 107.733, T2:107.733 to < 143.284, T3 ≥ 143.284); SII (T1 ≤ 391.057, T2:391.057 to <580.143, T3 ≥ 580.143); LMR (T1 ≤ 4.618, T2:4.618 to < 6.334, T3 ≥ 6.334).

Differences between continuous variables were analyzed using either a two-tailed Student’s *t* test or Mann–Whitney’s U test, while differences between categorical variables were assessed using either the Chi-square test or Fisher’s exact test. A logistic regression model was employed to identify independent factors influencing prognosis. The systemic immune inflammatory markers regression model served as the crude model, which was then adjusted in Model 1 to include age, gender, RNF213 p.R4810K variant, clinical phenotypes, Suzuki stage and surgical modality. Model 1 was further refined in Model 2 by incorporating hypertension, diabetes, hyperlipidemia, smoking, drinking, and homocysteine. Furthermore, the predictive performance of the model for long-term prognosis was evaluated by constructing a receiver operating characteristic (ROC) curve and calculating the area under the curve (AUC).

## Results

### Baseline characteristics of participants

The baseline characteristics of Moyamoya disease (MMD) patients are presented in Table 1 and Supplementary material. Our study ultimately included 851 cases of MMD patients, with 765 cases classified as having favorable long-term prognosis (89.9%) and 86 cases classified as having unfavorable long-term prognosis (10.1%). Included in the study were a total of 851 participants, consisting of 359 men (42.2%) and 492 women (57.8%). The median age of the individuals was 41 years, with a range of 34–48 years. The presence of the RNF213 p.R4810K variant was more prevalent in the group with favorable long-term outcomes compared to those with unfavorable outcomes (21.303% vs. 7.317%, *p* = 0.003). There is a statistically significant difference in the risk of adverse prognosis between patients with early and advanced stages of Suzuki stage (*p* = 0.009). Hypertension, as a common risk factor for stroke, was more prevalent in the unfavorable long-term prognosis group compared to the favorable long-term prognosis group (47.674% vs. 33.333%, *p* = 0.008). Patients in the unfavorable long-term prognosis group had higher levels of MONO, ALT, and TG compared to the favorable long-term prognosis group (*p* = 0.015, *p* = 0.025, *p* = 0.017). Significant differences in long-term prognosis outcomes were observed among patients in the TIA group (*p* = 0.017), while no significant differences were observed in the ischemic and hemorrhagic groups. There were no significant statistical differences in the prevalence of smoking history, alcohol consumption, diabetes, dyslipidemia, or hyperhomocysteinemia between the two groups. Additionally, there were no significant statistical differences in WBC, LY, NEUT, PLT, AST, Ca, P, CHO, HDL-C, LDL-C, ApoA1, ApoB, or Hcy levels between the two groups.

**Table 1:** Baseline characteristics of MMD.

Characteristics	Favorable prognosis (*n* = 765)	Unfavorable prognosis (*n* = 86)	*p*
Age, years, median [IQR]	41.000 [33.000, 48.000]	42.000 [35.000, 49.000]	0.068
Gender, male, *n* (%)	444 (58.039)	48 (55.814)	0.692
BMI, median [IQR]	25.195 [22.857, 27.778]	24.057 [22.039, 26.990]	0.102
RNF213 variant, *n* (%)	157 (21.303)	6 (7.317)	0.003**
Suzuki stage, *n* (%)			
1–3	425 (55.556)	35 (40.698)	0.009**
4–6	340 (44.444)	51 (59.302)	
History of risk factors, *n* (%)			
Hypertension	255 (33.333)	41 (47.674)	0.008**
Diabetes	83 (10.850)	8 (9.302)	0.660
Hyperlipidemia	72 (9.412)	7 (8.140)	0.700
Hyperhomocysteinemia	12 (1.569)	3 (3.488)	0.200
Alcohol drinking	76 (9.935)	8 (9.302)	0.852
Cigarette smoking	117 (15.294)	11 (12.791)	0.538
Clinical phenotypes, *n* (%)			0.017*
TIA	248 (32.418)	15 (17.442)	
Infarction	285 (37.255)	40 (46.511)	
Hemorrhage	232 (30.327)	31 (36.047)	
Surgical modality, *n* (%)			
Indirect revascularization	317(41.438)	37(43.023)	0.777
Direct/combined revascularization	448(58.562)	49(56.977)	
Follow-up time, median [IQR]	23[14, 40.5]	23.5[10, 53.25]	0.973
Follow-up events, *n* (%)	104 (13.595)	41 (47.674)	<0.001***
TIA	90 (11.765)	6 (6.976)	
Ischemia stroke	12 (1.569)	11 (12.791)	
Hemorrhage stroke	2 (0.261)	24 (27.907)	
Laboratory indicators, median [IQR]			
WBC,count, 10^9^/L	6.420 [5.360, 7.560]	6.430[5.460, 7.920]	0.468
LY, count, 10^9^/L	1.900 [1.570, 2.330]	1.880[1.560, 2.250]	0.358
MONO, count, 10^9^/L	0.350 [0.280, 0.440]	0.390[0.310, 0.470]	0.015*
NEUT, count, 10^9^/L	3.880 [3.070, 4.820]	4.000[3.290, 5.060]	0.079
PLT, count, 10^9^/L	239.000 [204.000, 277.000]	241.000[196.000, 273.000]	0.561
ALT, U/L	21.200 [14.000, 31.800]	24.000[16.900, 36.200]	0.025*
AST, U/L	18.200 [15.000, 23.000]	18.200[15.500, 24.700]	0.400
Calcium, mmol/L	2.410 [2.340, 2.480]	2.400[2.300, 2.470]	0.227
Phosphorus, mmol/L	1.150 [1.020, 1.280]	1.100[0.990, 1.270]	0.388
TG, mmol/L	1.250 [0.840, 1.740]	1.360[1.100, 1.980]	0.017*
CHO, mmol/L	4.210 [3.520, 4.820]	4.020[3.220, 4.710]	0.183
HDL-C, mmol/L	1.250 [1.050, 1.450]	1.240[1.110, 1.340]	0.885
LDL-C, mmol/L	2.350 [1.790, 2.980]	2.260[1.650, 2.780]	0.305
APOA1, g/L	1.280 [1.140, 1.460]	1.290[1.190, 1.460]	0.400
APOB, g/L	0.830 [0.690, 0.990]	0.810[0.620, 0.970]	0.238
Hcy, μmol/L	11.200 [8.410, 14.100]	11.700[8.500, 17.020]	0.217

BMI, Body mass index; TIA, Transient ischemic attack; WBC, White blood cell; LY, Lymphocutaneous; MONO, Monocytes; NEUT, Neutrophil; PLT, Platelet; ALT, Alanine aminotransferase; AST, Aspartate aminotransferase; TG, Triglyceride; CHO, Total cholesterol; HDL-C, High-density lipoprotein cholesterol; LDL-C, Low-density lipoprotein cholesterol; ApoA1, Apolipoprotein A1; ApoB, Apolipoprotein B; Hcy, Homocysteine.

*p < 0.05, **p < 0.01, and ***p < 0.001.

The overall median follow-up time was 23 [13, 42] months, with no significant statistical difference in median follow-up time between the favorable and unfavorable long-term prognosis groups (23 [14, 40.5] vs. 23.5 [10, 53.25], *p* = 0.268). The rate of stroke events during follow-up was significantly higher in the unfavorable long-term prognosis group compared to the favorable long-term prognosis group (47.674% vs. 13.595%, *p* < 0.001).

### Relationship between systemic immune-inflammatory marker levels and unfavorable long-term prognosis

**Figure 2:**
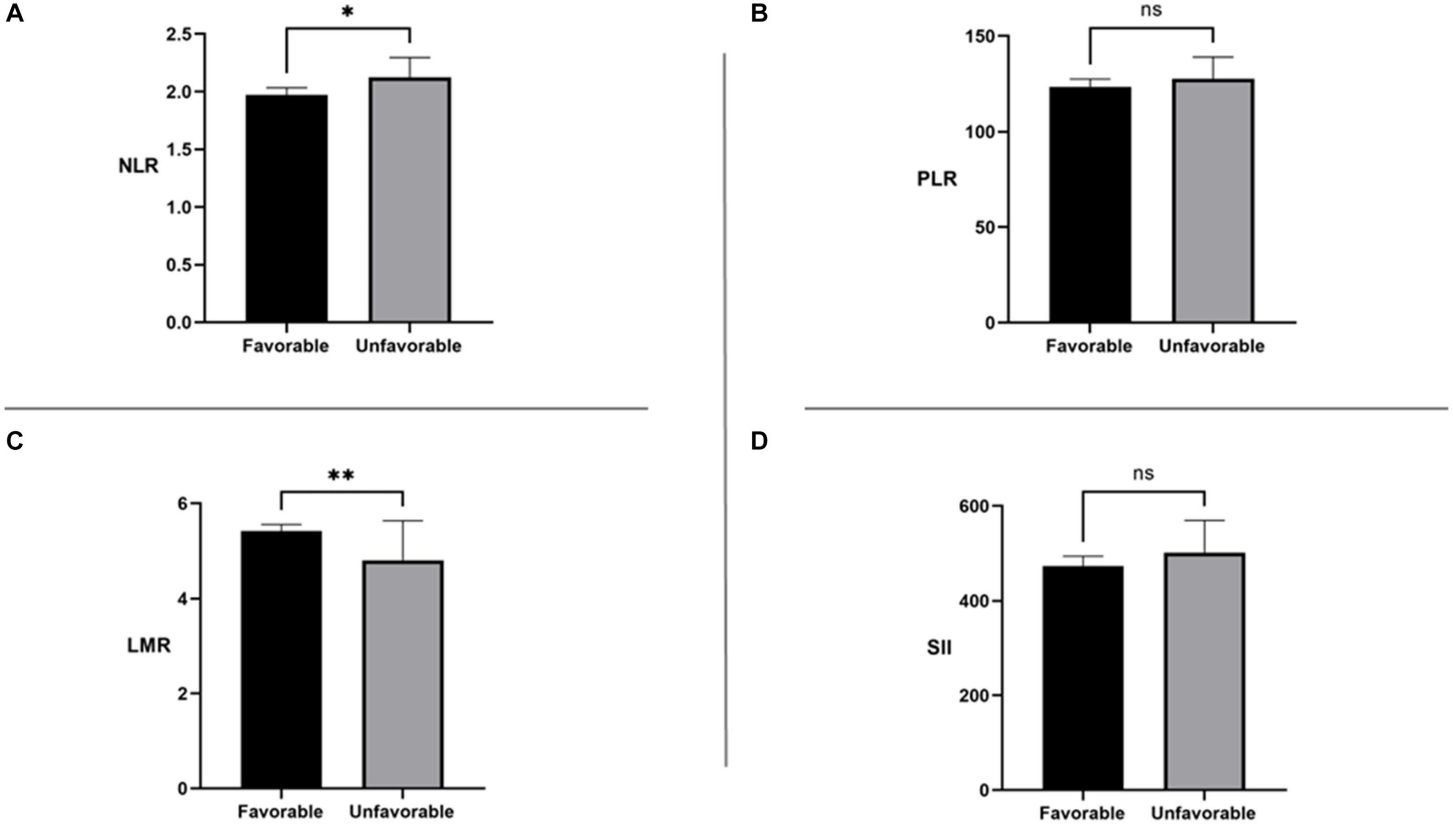
NLR: neutrophil-to-lymphocyte ratio; SII: systemic immune-inflammation index; PLR: platelet-to-lymphocyte ratio; LMR: lymphocyte-to-monocyte ratio. ns, No significance *p < 0.05 **p < 0.01.

Figure 2 shows the differences in NLR, PLR, SII, and LMR between the two groups. In the unfavorable long-term prognosis group, NLR levels were significantly higher than those in the favorable long-term prognosis group (2.113 [1.721, 2.913] vs. 1.973 [1.545, 2.510], *p* = 0.037), while LMR levels were lower than those in the favorable long-term prognosis group (4.800 [3.694, 6.094] vs. 5.420 [4.294, 6.917], *p* = 0.004). There were no significant differences in SII and PLR values between the two groups (*p* > 0.05). The differences between systemic immune inflammatory marker levels in the favorable and unfavorable prognosis groups are shown in Table 2. In the low-level LMR group, 41 individuals had a poor mid- and long-term prognosis, representing 47.674% of the group. The medium-level group had 25 people with an unfavorable long-term prognosis, accounting for 29.070%, while the high-level group had 20 individuals with an unfavorable long-term prognosis, making up 23.256%. The statistical analysis showed a significant difference between the groups (*p* = 0.009), indicating a strong association between LMR levels and long-term prognosis. Interestingly, there was no disparity in the risk of unfavorable long-term prognosis across the NLR, SII, and PLR groups, suggesting a lack of clear relationship between these immune-inflammatory markers and long-term prognosis.

**Table 2:** Baseline systemic immune-inflammatory markers in postoperative patients with MMD.

Characteristics	Favorable prognosis (*n* = 765)	Unfavorable prognosis (*n* = 86)	*p*
**NLR**			
Continuous	1.973 [1.545, 2.510]	2.113 [1.721, 2.913]	0.037*
Tertiles			
T1 ( ≤ 1.718)	262 (34.248)	21 (24.419)	0.184
T2 (1.718 to < 2.312)	252 (32.941)	32 (37.209)	
T3 ( ≥ 2.312)	251 (32.810)	33 (38.372)	
**PLR**			
Continuous	123.529 [99.565, 156.780]	125.000 [94.811, 164.762]	0.636
Tertiles			
T1 ( ≤ 107.733)	257 (33.595)	26 (30.233)	0.790
T2 (107.733 to <143.284)	255 (33.333)	29 (33.721)	
T3 ( ≥ 143.284)	253 (33.072)	31 (36.047)	
**SII**			
Continuous	473.199 [349.249, 661.333]	499.737 [377.849, 653.757]	0.141
Tertiles			
T1 ( ≤ 391.057)	259 (33.856)	24 (27.907)	0.519
T2 (391.057 to < 580.143)	252 (32.941)	32 (37.209)	
T3 ( ≥ 580.143)	254 (33.203)	30 (34.884)	
**LMR**			
Continuous	5.420 [4.294, 6.917]	4.800 [3.694, 6.094]	0.004**
Tertiles			
T1 ( ≤ 4.618)	242 (31.634)	41 (47.674)	0.009**
T2 (4.618 to < 6.334)	259 (33.856)	25 (29.070)	
T3 ( ≥ 6.334)	264 (34.510)	20 (23.256)	

NLR, Neutrophil-to-lymphocyte ratio; SII, Systemic immune-inflammation index; PLR, Platelet-to-lymphocyte ratio; LMR, Lymphocyte-to-monocyte ratio.

*p < 0.05.

**p < 0.01.

**Figure 3:**
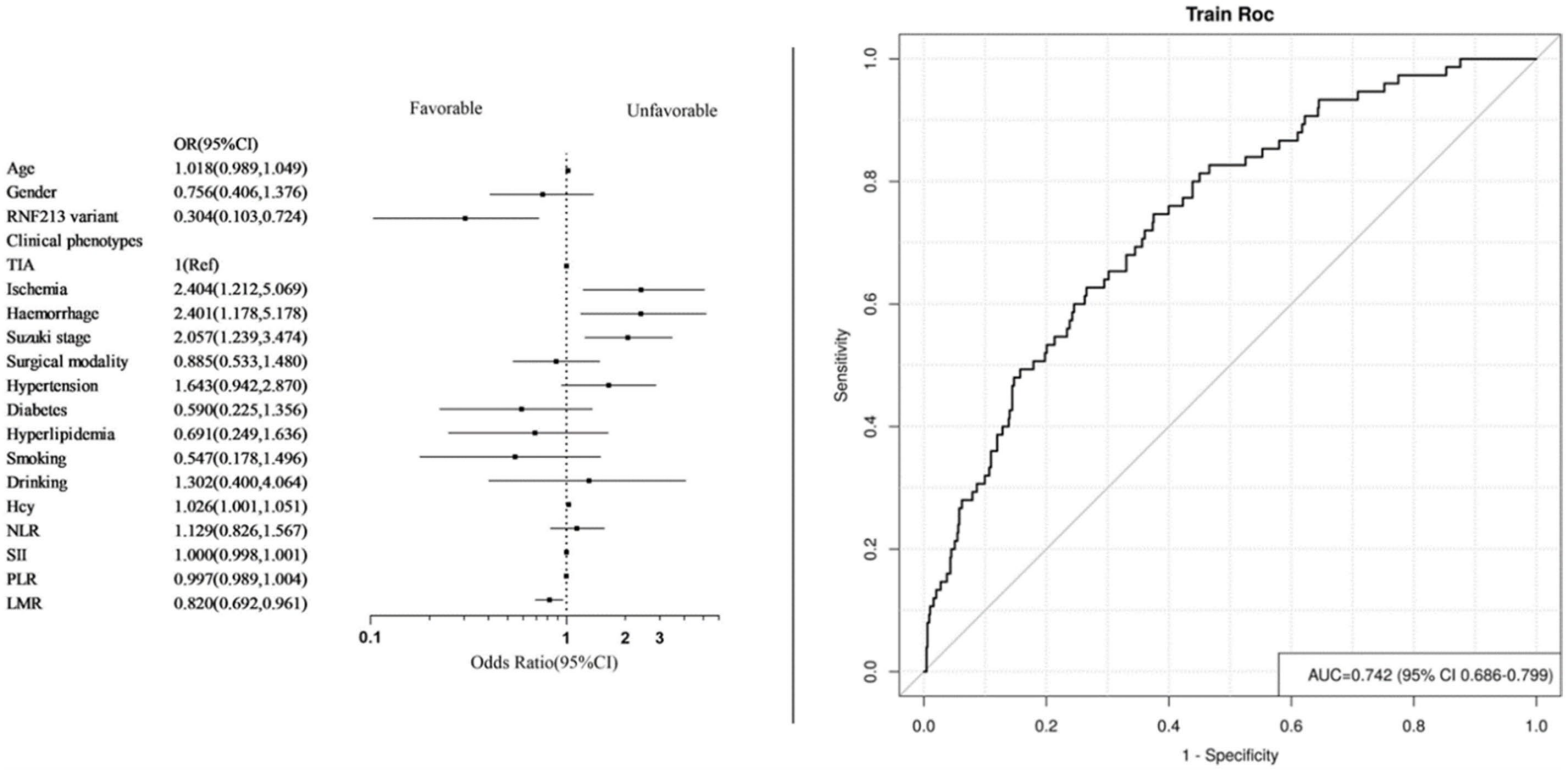
Forest plot and ROC curve of model 2. (Systemic immune inflammatory markers as continuous variables).

We performed univariate logistic regression analysis of systemic immune-inflammatory markers and some factors that may be associated with the risk of unfavorable prognosis (Tables 3, 4). Identify statistically significant factors and factors previously documented in the literature as being linked to poor prognosis, then conduct multifactor logistic regression analysis on them alongside systemic immune and inflammatory markers. Logistic regression analysis incorporated systemic immune and inflammatory markers as continuous variables (Table 5). The final adjusted model 2 indicated that elevated LMR levels are protective factors for long-term prognosis (OR = 0.820, 95% CI [0.692–0.961], *p* = 0.018), and patients with RNF213 mutations had a lower risk of unfavorable long-term prognosis compared to those without these mutations (OR = 0.301, 95% CI [0.102–0.707], *p* = 0.015). Additionally, homocysteine levels, clinical phenotypes, and Suzuki stage are associated with the risk of unfavorable long-term prognosis. No significant relationships were found between other systemic immune-inflammatory indicators and long-term prognosis. The AUC = 0.742 (95% CI [0.686–0.799]) (Figure 3).

**Table 3:** Univariate logistic regression of systemic immune-inflammatory markers.

Characteristics	OR(95%CI)	*p* value
**NLR**		
Continuous	1.178 [1.05, 1.321]	0.005**
Tertiles		
T1 (≤1.718)	Reference	-
T2 (1.718 to <2.312)	1.584 [0.890, 2.821]	0.118
T3 (≥2.312)	1.640 [0.924, 2.912]	0.091
**PLR**		
Continuous	1.001 [0.997, 1.005]	0.511
Tertiles		
T1 (≤107.733)	Reference	-
T2 (107.733 to < 143.284)	1.124 [0.644, 1.962]	0.681
T3 (≥143.284)	1.211 [0.699, 2.098]	0.494
**SII**		
Continuous	1.001 [1.0, 1.001]	0.028*
Tertiles		
T1 (≤391.057)	Reference	-
T2 (391.057 to <580.143)	1.370 [0.785, 2.392]	0.268
T3 (≥580.143)	1.275 [0.725, 2.240]	0.399
**LMR**		
Continuous	0.835 [0.738, 0.945]	0.004**
Tertiles		
T1 (≤4.618)	Reference	-
T2 (4.618 to <6.334)	0.570 [0.336, 0.965]	0.037*
T3 (≥6.334)	0.447 [0.255, 0.785]	0.005**

OR, Odds ratio; CI, Confidence interval; MMD, Moyamoya disease; NLR, Neutrophil-to-lymphocyte ratio; SII, Systemic immune-inflammation index; PLR, Platelet-to-lymphocyte ratio; LMR, Lymphocyte-to-monocyte ratio.

*p < 0.05.

**p < 0.01.

**Table 4:** Univariate logistic regression of relevant risk factors.

Characteristics	OR (95%CI)	*p* value
Age	1.023 [1.0, 1.048]	0.053
Gender	1.095 [0.699, 1.716]	0.692
RNF213pR4810K	0.292 [0.125, 0.682]	0.004**
BMI	0.944 [0.872, 1.021]	0.149
Suzuki stage		
1–3	Reference	-
4–6	1.821 [1.158, 2.866]	0.010*
Clinical phenotypes		
TIA	Reference	-
Infarction	2.320 [1.252, 4.302]	0.008**
Hemorrhage	2.209 [1.163, 4.198]	0.016*
Hypertension	1.822 [1.163, 2.855]	0.009**
Diabetes	0.843 [0.393, 1.806]	0.660
Hyperlipidemia	0.853 [0.379, 1.917]	0.700
Smoking	0.812 [0.419, 1.576]	0.539
Alcohol drinking	0.930 [0.433, 1.999]	0.852
Surgical modality		
Indirect revascularization	Reference	-
Direct/combined revascularization	0.937 [0.597, 1.470]	0.777
ALT	1.003 [0.997, 1.01]	0.311
AST	1.008 [0.992, 1.025]	0.332
Ca	0.345 [0.048, 2.5]	0.292
P	0.821 [0.288, 2.335]	0.711
TG	1.175 [0.941, 1.467]	0.154
CHO	0.837 [0.644, 1.089]	0.185
HDL-C	1.253 [0.572, 2.746]	0.573
LDL-C	0.862 [0.638, 1.165]	0.335
APOA1	1.232 [0.675, 2.248]	0.496
APOB	0.792 [0.402, 1.56]	0.500
Hcy	1.021 [1.0, 1.042]	0.047*

*p < 0.05.

**p < 0.01.

**Table 5:** Association of systemic immune-inflammatory markers and long-term prognosis with MMD.

Characteristics	Crude model		Model 1		Model 2	
	OR (95% CI)	*p* value	OR (95% CI)	*p* value	OR (95% CI)	*p* value
**NLR**						
Continuous	1.148 (0.852, 1.577)	0.371	1.108 (0.817, 1.529)	0.510	1.129 (0.826, 1.567)	0.447
Tertiles						
T1 (≤1.718)	Reference		Reference		Reference	
T2 (1.718 to < 2.312)	1.496 (0.733, 3.091)	0.272	1.548 (0.735, 3.311)	0.254	1.721 (0.776, 3.909)	0.186
T3 (≥2.312)	1.500 (0.599, 3.760)	0.386	1.330 (0.500, 3.532)	0.567	1.521 (0.537, 4.318)	0.430
**PLR**						
Continuous	0.997 (0.990, 1.003)	0.345	0.996 (0.989, 1.004)	0.333	0.997 (0.989, 1.004)	0.412
Tertiles						
T1 (≤107.733)	Reference		Reference		Reference	
T2 (107.733 to < 143.284)	1.031 (0.558, 1.906)	0.923	1.004 (0.524, 1.922)	0.847	1.281 (0.640, 2.591)	0.486
T3 (≥143.284)	1.006 (0.484, 2.087)	0.987	0.996 (0.456, 2.171)	0.991	1.192 (0.522, 2.741)	0.677
**SII**						
Continuous	1.000 (0.998, 1.002)	0.991	1.000 (0.998, 1.002)	0.913	1.000 (0.998, 1.001)	0.790
Tertiles						
T1 (≤391.057)	Reference		Reference		Reference	
T2 (391.057 to < 580.143)	0.926 (0.448, 1.928)	0.837	0.927 (0.438, 1.979)	0.844	0.911 (0.408, 2.056)	0.821
T3 (≥580.143)	0.661 (0.241, 1.813)	0.420	0.703 (0.245, 2.025)	0.512	0.551 (0.179, 1.694)	0.297
**LMR**						
Continuous	0.856 (0.738, 0.982)	0.032	0.835 (0.711, 0.969)	0.022	0.820 (0.692, 0.961)	0.018
Tertiles						
T1 (≤4.618)	Reference		Reference		Reference	
T2 (4.618 to <6.334)	0.548 (0.310, 0.955)	0.036	0.488 (0.265, 0.879)	0.018	0.508 (0.264, 0.954)	0.038
T3 (≥6.334)	0.453 (0.234, 0.856)	0.016	0.400 (0.196, 0.794)	0.010	0.433 (0.204, 0.893)	0.026

OR, Odds ratio; CI, Confidence interval; MMD, Moyamoya disease; NLR, Neutrophil-to-lymphocyte ratio; SII, Systemic immune-inflammation index; PLR, Platelet-to-lymphocyte ratio; LMR, Lymphocyte-to-monocyte ratio.

Crude model includes NLR, SII, PLR, and LMR.

Model 1 was adjusted for age, gender, RNF213 p.R4810K variant, Clinical phenotypes, Suzuki stage, and surgical modality.

Model 2 was adjusted for all the variables in model 1 plus hypertension, diabetes, hyperlipidemia, smoking, drinking, and homocysteine.

**Figure 4:**
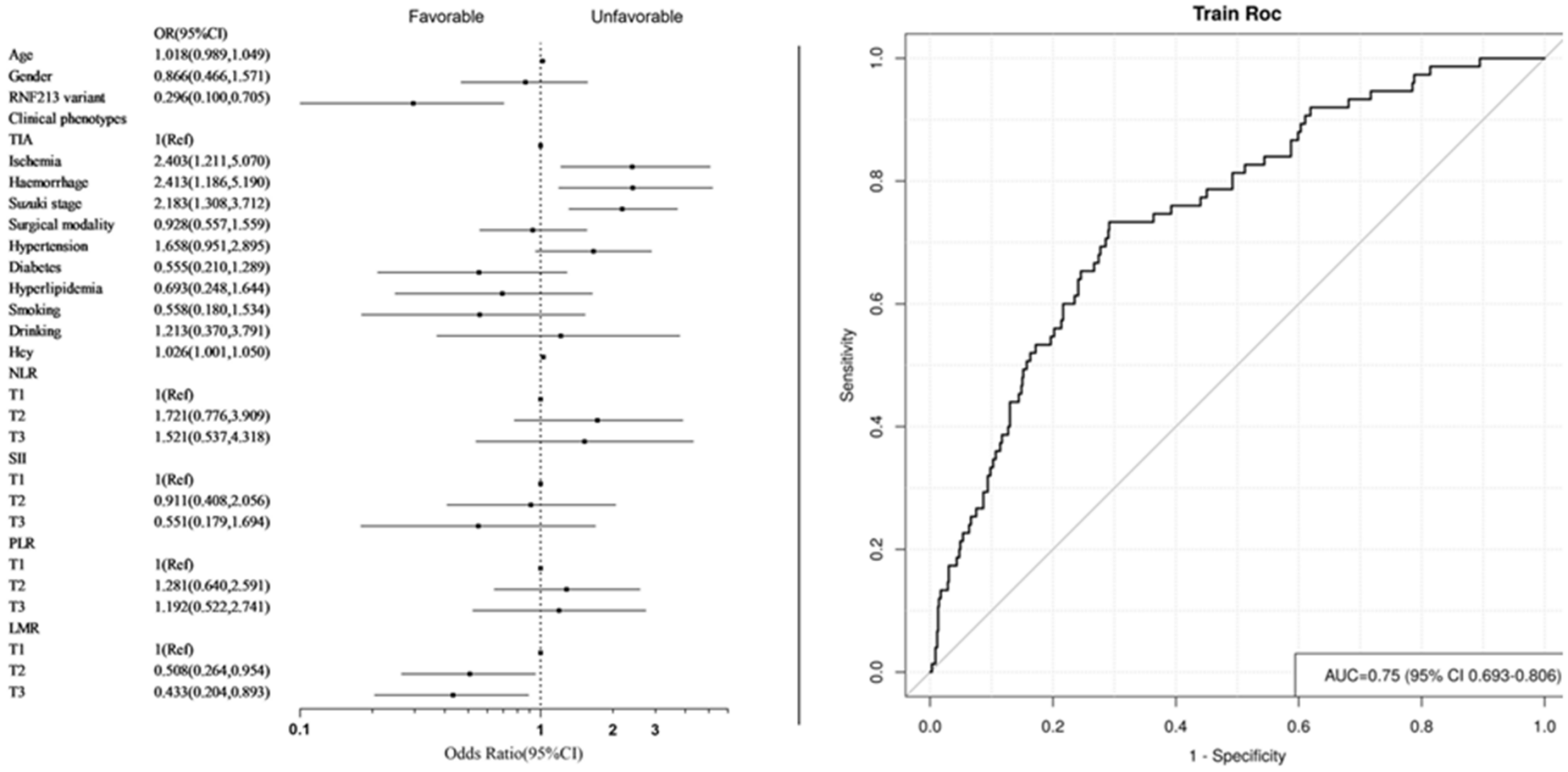
Forest plot and ROC curve of model 2. (Systemic immune inflammation markers as categorical variables).

To address individual variability in systemic immune and inflammatory markers derived from blood tests, these markers were categorized into low (T1), medium (T2), and high levels (T3) for further analysis (Table 5). The final adjusted model 2 results revealed that compared with the T1 group, the increased LMR levels in the T3 and T2 groups are protective factors for long-term prognosis (T2: OR = 0.508, 95% CI [0.264–0.954], *p* = 0.038; T3: OR = 0.433, 95% CI [0.204–0.893], *p* = 0.026). The AUC = 0.750 (95% CI [0.693–0.806]) (Figure 4).

## Discussion

The etiology and pathogenesis of Moyamoya disease (MMD) remain unclear and are widely regarded as a multifactorial disease resulting from genetic, immune, inflammatory, and other factors. The primary treatment for MMD revolves around surgical intervention, with cerebral revascularization being the mainstay. Patients undergoing cerebral revascularization surgery experience a significant reduction in the risk of stroke occurrence (4). The long-term prognosis of patients with Moyamoya disease (MMD) after surgery is challenging to predict. Previous research has attempted to establish long-term prognosis prediction models based on genetic factors, clinical characteristics, and nutritional indices (6, 7, 9). However, insufficient consideration has been given to the impact of systemic immune-inflammatory factors on long-term prognosis.

This study included 851 subjects who underwent cerebral revascularization surgery and found significant differences in neutrophil-to-lymphocyte ratio (NLR) and lymphocyte-to-monocyte ratio (LMR) between the groups with favorable and unfavorable long-term prognosis. NLR levels were higher in the unfavorable long-term prognosis group compared to the favorable group, while LMR levels were lower in the unfavorable long-term prognosis group. However, systemic immune-inflammatory index (SII) and platelet-to-lymphocyte ratio (PLR) showed no significant differences between the two groups. Logistic regression analysis revealed that LMR serves as an independent risk factor in predicting unfavorable long-term prognosis. Furthermore, as the level of LMR increases, the risk of unfavorable long-term prognosis decreases. No significant relationship was observed between other inflammation markers and long-term prognosis. Therefore, LMR is considered a novel indicator for predicting long-term prognosis after cerebral revascularization surgery in Moyamoya disease patients. The lymphocyte-to-monocyte ratio (LMR) is easily obtainable in clinical practice and has been widely used as a biomarker for predicting patient outcomes in various fields including cerebrovascular diseases, cancer, and neurology (12, 15–18). All suggest that lower LMR levels are indicative of worse long-term outcomes in patients, although the specific mechanisms behind this remain unclear.

In recent years, there has been a growing focus on neuroinflammation, with numerous studies demonstrating the significant impact of inflammatory processes on the initiation and progression of ischemic stroke (19–22). It is believed that lymphocyte counts have a neuroprotective effect and can enhance neurological function, while monocytes and neutrophils may exacerbate symptoms (23–25). Research has indicated that ischemia of the brain has the potential to inhibit the functioning of the lymphatic system. Consequently, this can elevate the likelihood of infections occurring after a stroke, which ultimately serves as a leading contributor to the mortality rate among individuals who have suffered a stroke (26). During the acute phase of ischemic stroke, the inflammatory response plays a dual role by inducing cell death and tissue damage while also triggering a preconditioning state in the brain to exert self-protective effects. Inflammatory mediators automatically restrain the progression of pathological processes and facilitate the repair of damaged tissues (27). The physiological reactions post-stroke may be ascribed to the extravasation of leukocytes and the discharge of various inflammatory agents, setting off a cascade of events that culminates in neuronal demise or programmed cell death, leading to unfavorable outcomes for stroke sufferers (28). An increase in lymphocyte levels can elevate anti-inflammatory proteins such as cytokine interleukin (IL)-10, while simultaneously inhibiting the expression of pro-inflammatory proteins like IL-6 and tumor necrosis factor-alpha (TNF-α), thereby producing neuroprotective effects (29, 30). Post-stroke systemic stress leads to a decrease in lymphocyte count, promoting the activation of the renin-angiotensin system, resulting in cortisol release, which induces lymphocyte apoptosis (31). A dip in lymphocyte levels post-acute ischemic stroke is linked to postponed brain harm and deteriorated functional results, ultimately resulting in adverse outcomes for stroke patients (32–34). However, our research discovered that there was no noteworthy variance in lymphocyte count between the group exhibiting favorable long-term prognosis and the group showing unfavorable long-term prognosis. On the other hand, the monocyte count was markedly elevated in the group with unfavorable long-term prognosis when juxtaposed with the group with favorable long-term prognosis, leading to a conspicuous contrast in LMR levels. This disparity in LMR between the two groups may be primarily attributed to the aforementioned factor.

Monocytes, serving as prime contributors to systemic inflammatory reactions, are classified into three principal groupings in the human populace and fulfill distinct roles in the pathophysiology of ischemia (35). In human, monocytes are regrouped in three main subsets based on their CD14 and CD16 expression levels, which are the classical subset (CD14^++^CD16^−^), the intermediate subset (CD14^++^CD16^+^), and the non-classical subset (CD14^+^CD16^++^). CD14^++^CD16^−^ monocytes, identified by elevated CCR2 chemokine receptor levels and low CX3CR1 expression, add to inflammation by discharging cytokines like IL-1β, IL-6, and TNF-α, while CD14^+^CD16^++^ monocytes counteract inflammation by producing IL-10 (36). In the aftermath of a stroke event, an upsurge in classical monocytes is an autonomous forecaster of unfavorable outcomes at the three-month mark, whereas a greater proportion of non-conventional monocytes is linked to improved outcomes following the onset of acute ischemic stroke (37). Furthermore, there is a positive relationship between total monocyte counts and mRS scores at the three-month point, indicating that higher monocyte counts are related to unfavorable functional prognosis (38).

Moyamoya disease (MMD) is an important etiology of stroke in young adults in Southeast Asia. Therefore, integrating lymphocyte and monocyte counts into the lymphocyte-to-monocyte ratio (LMR) as a novel predictor of long-term prognosis after surgical treatment for MMD is highly meaningful. Our study revealed, for the first time, that LMR levels are independent risk factors for unfavorable long-term prognosis. Decreased LMR levels often lead to worse outcomes.

Nonetheless, our research encountered several constraints. Primarily, it was a retrospective investigation carried out at a sole research facility, exclusively involving grown-ups while omitting minors. This could potentially impede its effectiveness and introduce biases in selection. Secondly, we only calculated systemic immune-inflammatory markers at admission without dynamic monitoring and observation, which may affect the correlation between LMR in MMD patients and long-term prognosis. Thirdly, we only used the modified Rankin Scale (mRS) to assess functional outcomes, which may not provide a detailed definition of favorable and unfavorable long-term prognosis.

## Conclusion

Systemic immune and inflammatory markers that can be easily obtained in a clinical setting play a crucial role in predicting the risk of Moyamoya disease and post-surgery prognosis. Our study shows that lymphocyte-to-monocyte ratio (LMR) levels are independently linked to an increased risk of unfavorable long-term prognosis, highlighting LMR as a new and effective predictor for postoperative moyamoya patients. It is essential to conduct future prospective studies with larger, multicenter cohorts to validate our results.

## Data Availability

The raw data supporting the conclusions of this article will be made available by the authors, without undue reservation.
