# Supplementary material for: Systemic immune-inflammatory markers and long-term prognosis after revascularization in Moyamoya disease: a retrospective study
Source: Front Neurol. 2024 Sep 2;15:1418729. doi: 10.3389/fneur.2024.1418729 (PMC11402733; doi:10.3389/fneur.2024.1418729)
Supplement: Supplementary file 1 [file Data_Sheet_1.docx]

Supplementary material. Baseline characteristics of MMD.

| Characteristics | ALL(n=851) | Favorable prognosis(n=765) | Unfavorableprognosis (n=86) | P |
| --- | --- | --- | --- | --- |
| Age ,y，median[IQR] | 41.000[34.000,48.000] | 41.000[33.000,48.000] | 42.000[35.000,49.000] | 0.068 |
| Gender ,male，n(%) | 492(57.814) | 444(58.039) | 48(55.814) | 0.692 |
| BMI ,median[IQR] | 25.076[22.833,27.757] | 25.195[22.857,27.778] | 24.057[22.039,26.990] | 0.102 |
| RNF213 variant ,n(%) | 163(19.902) | 157(21.303) | 6(7.317) | 0.003** |
| Suzuki stage,n(%) |  |  |  |  |
| 1-3 | 460(54.054) | 425(55.556) | 35(40.698) | 0.009** |
| 4-6 | 391(45.946) | 340(44.444) | 51(59.302) |  |
| History of risk factors，n（%） |  |  |  |  |
| Hypertension | 296(34.783) | 255(33.333) | 41(47.674) | 0.008** |
| Diabetes | 91(10.693) | 83(10.850) | 8(9.302) | 0.660 |
| Hyperlipidemia | 79(9.283) | 72(9.412) | 7(8.140) | 0.700 |
| Hyperhomocysteinemia | 15(1.763) | 12(1.569) | 3(3.488) | 0.200 |
| Alcohol drinking | 84(9.871) | 76(9.935) | 8(9.302) | 0.852 |
| Cigarette Smoking | 128(15.041) | 117(15.294) | 11(12.791) | 0.538 |
| Clinical phenotypes,n(%) |  |  |  | 0.017* |
| TIA | 263(30.905) | 248（32.418） | 15（17.442） |  |
| Infarction | 325(38.190) | 285（37.255） | 40（46.511) |  |
| Hemorrhage | 263(30.905) | 232（30.327） | 31（36.047) |  |
| Surgical modality ,n(%) |  |  |  |  |
| Indirect revascularization | 354(41.598) | 317(41.438) | 37(43.023) | 0.777 |
| Direct/combined revascularization | 497(58.402) | 448(58.562) | 49(56.977) |  |
| Follow-up Time ,median[IQR] | 23[13,42] | 23[14,40.5] | 23.5[10,53.25] | 0.973 |
| Follow-up Events,n(%) | 145（17.039） | 104（13.595） | 41（47.674） | <0.001*** |
| TIA | 96（11.281） | 90（11.765） | 6（6.976） |  |
| Ischemia stroke | 23（2.703） | 12（1.569） | 11（12.791） |  |
| Haemorrhage stroke | 26（3.055） | 2（0.261） | 24（27.907） |  |
| Laboratory indicators,median[IQR] |  |  |  |  |
| WBC,count,10^9^/L | 6.420[5.360,7.590] | 6.420[5.360,7.560] | 6.430[5.460,7.920] | 0.468 |
| Lymphocyte,count,10^9^/L | 1.900[1.560,2.310] | 1.900[1.570,2.330] | 1.880[1.560,2.250] | 0.358 |
| Monocytes,count,10^9^/L | 0.360[0.290,0.440] | 0.350[0.280,0.440] | 0.390[0.310,0.470] | 0.015* |
| Neutrophil,count,10^9^/L | 3.910[3.100,4.860] | 3.880[3.070,4.820] | 4.000[3.290,5.060] | 0.079 |
| Platelet,count,10^9^/L | 239.000[202.000,277.000] | 239.000[204.000,277.000] | 241.000[196.000,273.000] | 0.561 |
| ALT,U/L | 21.700[14.100,32.100] | 21.200[14.000,31.800] | 24.000[16.900,36.200] | 0.025* |
| AST,U/L | 18.200[15.100,23.100] | 18.200[15.000,23.000] | 18.200[15.500,24.700] | 0.400 |
| Calcium,mmol/L | 2.410[2.340,2.480] | 2.410[2.340,2.480] | 2.400[2.300,2.470] | 0.227 |
| Phosphorus,mmol/L | 1.150[1.020,1.280] | 1.150[1.020,1.280] | 1.100[0.990,1.270] | 0.388 |
| Triglyceride, mmol/L | 1.260[0.860,1.770] | 1.250[0.840,1.740] | 1.360[1.100,1.980] | 0.017* |
| Total cholesterol, mmol/L | 4.200[3.500,4.810] | 4.210[3.520,4.820] | 4.020[3.220,4.710] | 0.183 |
| HDL-C, mmol/L | 1.250[1.060,1.450] | 1.250[1.050,1.450] | 1.240[1.110,1.340] | 0.885 |
| LDL-C, mmol/L | 2.340[1.770,2.950] | 2.350[1.790,2.980] | 2.260[1.650,2.780] | 0.305 |
| APOA1, g/L | 1.290[1.150,1.460] | 1.280[1.140,1.460] | 1.290[1.190,1.460] | 0.400 |
| APOB, g/L | 0.822[0.680,0.990] | 0.830[0.690,0.990] | 0.810[0.620,0.970] | 0.238 |
| Homocysteine, μmol/L | 11.200[8.410,14.320] | 11.200[8.410,14.100] | 11.700[8.500,17.020] | 0.217 |

BMI:body mass index; TIA:transient ischemic attack; WBC: white blood cell; LY: lymphocutaneous; MONO: monocytes; NEUT:neutrophil; PLT: platelet; ALT: alanine aminotransferase; AST: aspartate aminotransferase;TG:triglyceride; CHO: Total cholesterol; HDL-C:high-density lipoprotein cholesterol; LDL-C: low-density lipoprotein cholesterol; ApoA1, apolipoprotein A1; ApoB, apolipoprotein B; Hcy: Homocysteine.

*p<0.05,**p<0.01,***p<0.001.
